# Supplementary material for: Heparin–Superparamagnetic Iron Oxide Nanoparticles for Theranostic Applications
Source: Molecules. 2022 Oct 21;27(20):7116. doi: 10.3390/molecules27207116 (PMC9611043; doi:10.3390/molecules27207116)
Supplement: Supplementary file 1 [file molecules-27-07116-s001.zip › molecules-1932338-supplementary.pdf]

Supplementary Information
Heparin-Superparamagnetic Iron Oxide Nanoparticles for Theranostic Applications

1. LMWH (DALTEPARIN) synthesis and characterization.

LMWH was obtained via nitrous acid depolymerisation and reduction with $NaBH_4$: 2.0 g of unfractionated porcine mucosa heparin were dissolved in 33 mL H_2O, 33.3 mg ($NaNO_2$) were added to the reaction medium (pH =5.7). pH was brought to 2.4 with HCl 2N under stirring to generate nitrous acid (reaction was observed to start at pH =3 by the formation of N_2 bubbles) and continued for 30 minutes. After 30 minutes HCl was neutralized with NaOH 2M to a pH of 7 and 90 mg $NaBH_4$ were added to the reaction flask and stirred for 2 hours. Reaction was quenched by bringing the pH to 3 thus destroying residual reducing agent. A solution of 100 mL EtOH and 4.5% w/w CH_3COONa is prepared and a fractional separation with 32 and 54% EtOH/H_2O followed by two ultracentrifugations at 23,000 rpm x 10 min is carried to concentrate the fraction at lower weights obtaining an LMWH with a MW of 5,200 Da (analysed via TDA). The average MW and the polydispersity of LMWHs were evaluated with Size Exclusion Chromatography (SEC), coupled with triple detector array (TDA). LMWH obtained by oxidative deamination was characterized via a triple detector array obtaining a MW of 5,428 Da and a degree of molecular dispersion (MW/Mn) of 1,239 [63].

1.1 DA-LMWH NMR characterization

NMR two-dimensional homo- and hetero-nuclear single and multiple correlation techniques were used. **Fig. S1** shows the superposition of the aliphatic/carbohydrate areas of the COSY (red) and TOCSY homonuclear correlation spectra (blue). In the spectral zone relative to the heparin signals compared to the parent heparin, small variations of the chemical shifts of the signals of the I2S residue are observed at the level of the protons in position 2, which are not justified by desulfation thereof.

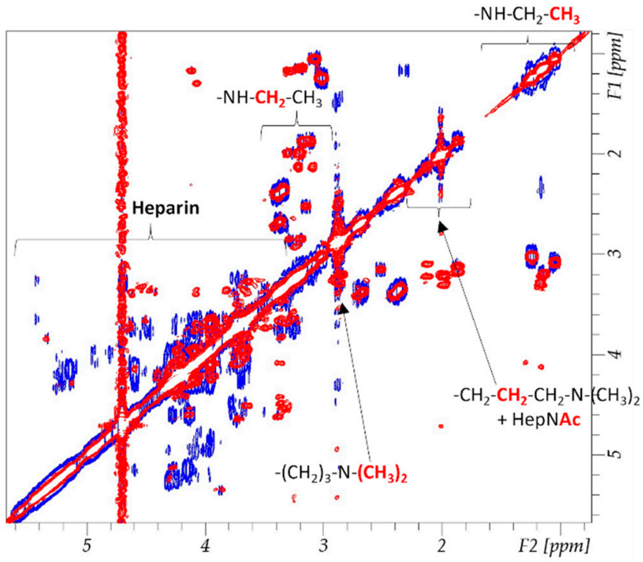

Figure S1. Superposition of the aliphatic / carbohydrate areas of the COSY (red) and TOCSY homonuclear correlation spectra (blue). Between 3.2 and 1.0 ppm=signals due to the aliphatic chain (EDC).

Heteronuclear spectra of **Figure S2** show the area of the anomeric signals and highlights that the signals of non-sulfated DA LMWH uronic acids do not undergo synthesis-induced changes compared to those of parent heparin. As well as the anomeric signals of glucosamine (5.4 ppm) do not appear significantly modified, as suggested by the homonuclear correlation spectra, too.

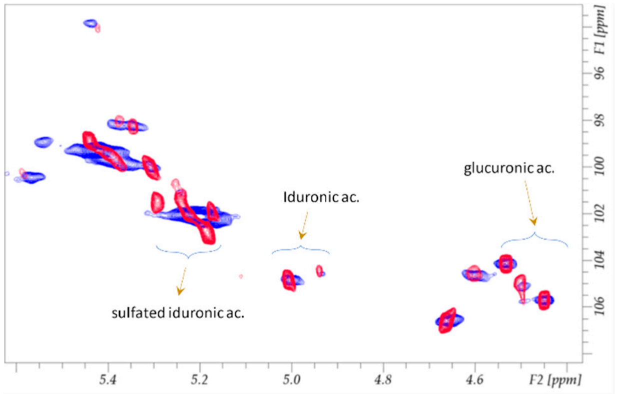

Figure S2. HSQC NMR – Expansion of heparin anomeric signals. Parent heparin: blue, DA-LMWH: red.

2. DA-BSA characterization

After coupling, the synthetic adduct DA-BSA was characterized via MALDI-TOF to obtain the degree of substitution. Assuming that the linkage involves DA as a monomer, the degree of substitution corresponded to the medium average derivatization of 50 DA per BSA (Δ=7,600) **(Fig. S3).** After optimization of the synthetic procedure, involving the reduction of DA and EDC by 2/3, the substitution degree was brought to 28 DA/BSA (Δ=4,200) **(Fig. S4).**

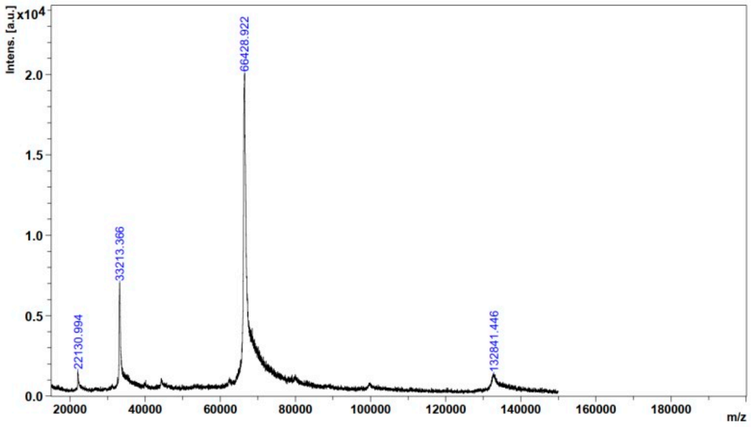

Figure S3. MALDI-TOF spectrum of uncoupled BSA.

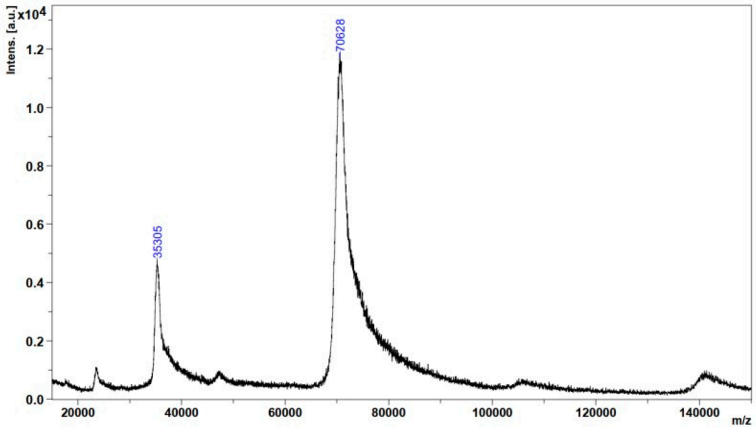

Figure S4. MALDI-TOF spectrum of coupled DA-BSA

3. FT-IR analyses of LMWH@SPION.

As shown in **Fig. S5** and **S6,** the functionalisation of the SPION3 and 4 systems is confirmed by their FT-IR spectra. The BSA and heparin profiles can be identified in SPION3, while heparin profile alone is present in SPION4. In advance, some diagnostic waves in SPION3 are 584 cm^{-1} (Fe-O); 1445 and 1406 cm^{-1} (bidentate ligand DA bound to Fe); 1541 cm^{-1} (aromatic sym C-C stretching) while in SPION4 are 583 cm^{-1} (Fe-O); 1438 and 1384 cm^{-1} (bidentate ligand DA bound to Fe); 1545 cm^{-1} (aromatic sym C-C stretching).

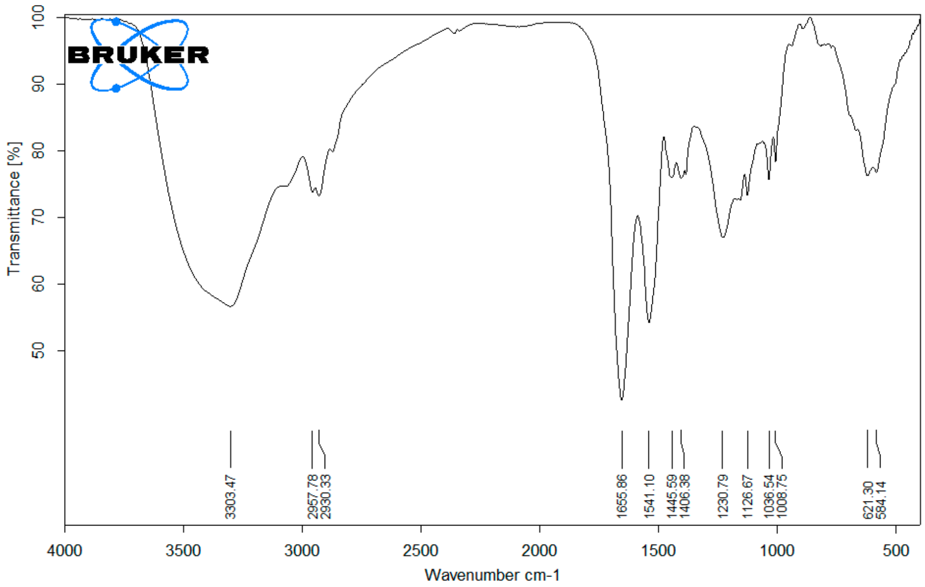

Figure S5. FT-IR spectrum of SPION3.

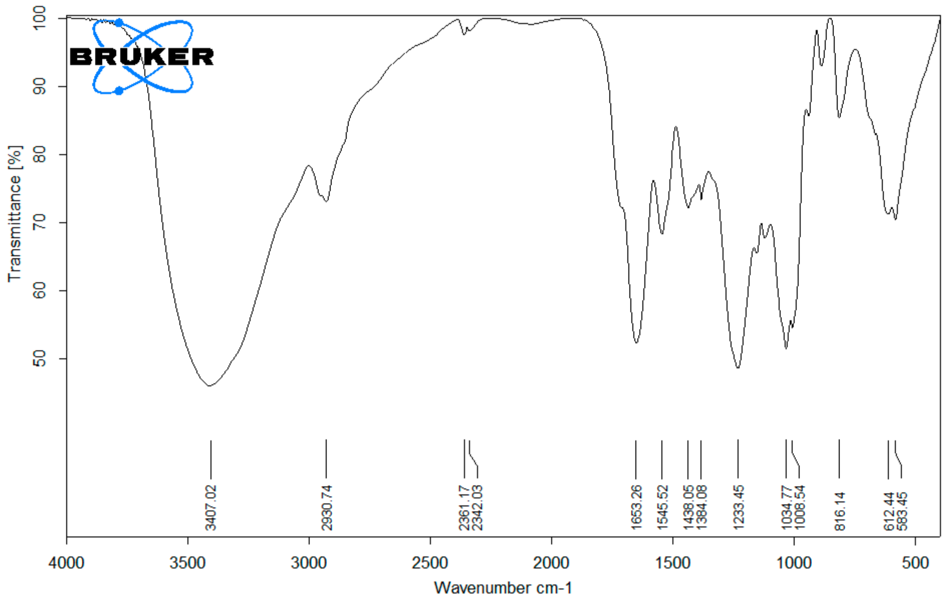

Figure S6. FT-IR spectrum of SPION4.

4. PTX release from SPION

Once the ability of the system to include the drug has been tested, the capability of releasing the drug was checked via a dialysis-based assay. PTX release was tested using a 3,500 kDa MWCO dialysis membrane; 3 mL of SPION3+PTX and SPION4+PTX were dialyzed against 35 mL of a 30:70 MeOH/HPLC grade H_2O solution. 20 µL of the extra dialysis were sampled at 0.25, 0.5, 1, 2, 4, 6, 24, 30, 48, 72, 88, 144, 168 and 240 h. Mobile phase was composed of acetonitrile: water (60:40). The UV-detection wavelength was set at λ = 230 nm, PTX was eluted at 6.3 min with a flow = 1 mL/min. Linear range of concentration used for the calibration curve (R^2 = 0.9978) is from 6 mg/L to 100 mg/L (6, 13, 25, 50, 100 mg/L), [64]. SPION3+PTX and SPION4+PTX kinetic release profiles appear quite different (**Fig S7** and **S8**).

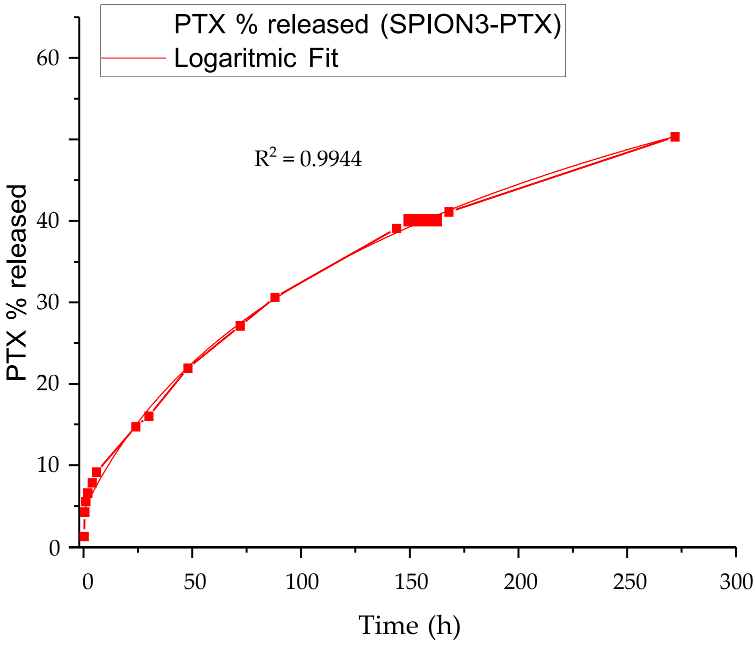

Figure S7. PTX release from system SPION3+PTX.

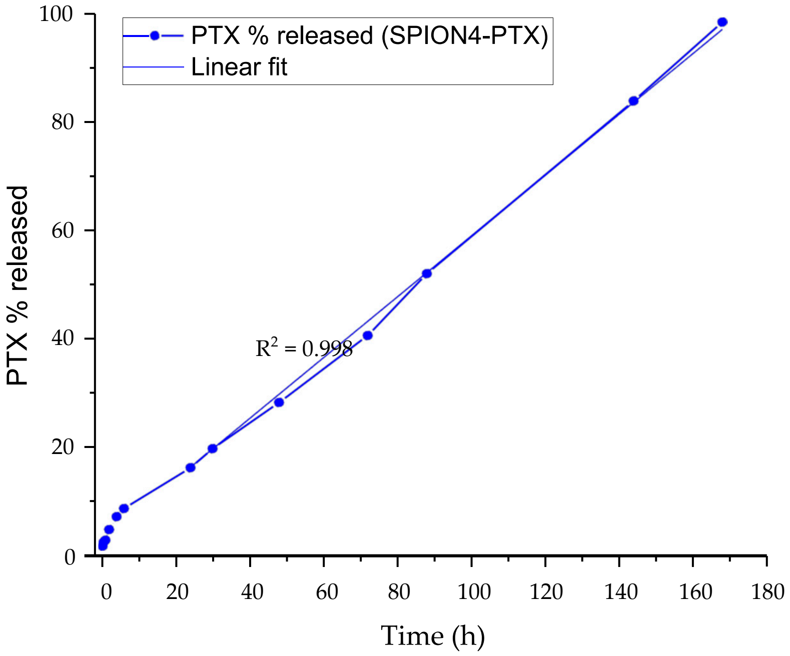

Figure S8. PTX release profile from SPION4-PTX.
